# P-904. Implementation of PEN-FAST: a Clinical Decision-Making Tool for Delabeling Penicillin Allergy in Low-Risk Patients

**DOI:** 10.1093/ofid/ofaf695.1110

**Published:** 2026-01-11

**Authors:** Priya Tyagaraj, Gwang-Yee Jessica Hu

**Affiliations:** Robert Wood Johnson University Hospital- Somerset, Somerville, New Jersey; Ernest Mario School of Pharmacy at Rutgers University, North Brunswick Township, NJ

## Abstract

**Background:**

Penicillin allergies are frequently reported but unconfirmed, leading to challenges for antimicrobial stewardship. Accurate reports of allergy manifestations can improve patient outcomes. PEN-FAST is a validated tool that identifies low-risk penicillin allergies accurately for delabeling without formal skin testing. This study aims to utilize PEN-FAST to identify low-risk patients eligible for delabeling based solely on an oral challenge, minimizing inappropriate broad-spectrum antibiotic use.

**Methods:**

This multi-center, two-phase, IRB-approved cohort study included adult patients with a documented penicillin allergy between January 2024 and April 2025 and excluded patients with any unconfirmed or anaphylactic allergy histories. Retrospectively, PEN-FAST scores, antibiotic use and allergy manifestations were assessed by chart review. Prospectively, eligible patients with PENFAST scores less than three received an oral amoxicillin challenge with monitoring of vital signs and hypersensitivity reactions. The primary outcome was the incidence of allergy delabeling, with secondary outcomes including continued use of broad-spectrum antibiotics after index admission delabeling, and the incidence of immune-mediated reactions post-oral challenge.

**Results:**

A total of 440 patients were screened retrospectively with 405 included for review. Of these, 310 patients (71%) had an unspecified penicillin allergy label, most often of unknown origin (36%), and 179 patients (44%) received broad-spectrum antibiotics, 139 (78%) of which received a cephalosporin. Only 20 (5%) patients had sufficient data to calculate PEN-FAST scores.

Prospectively, 305 patients were screened, and 35 met inclusion criteria. Twelve patients underwent an oral penicillin challenge, two of which experienced an immune mediated reaction requiring treatment and 11 others were delabeled based on non-immune mediated allergy histories. None of the patients who were delabeled were readmitted.

**Conclusion:**

Majority of the patients undergoing the oral challenge were successfully delabeled with no immune reactions. PEN-FAST use can help identify low-risk patients and support safe oral challenges, enabling the future use of penicillin-based antibiotics.

**Disclosures:**

All Authors: No reported disclosures

